# P-1168. Pharmacokinetics, Safety, and Tolerability of Funobactam in Combination with Imipenem/Cilastatin in Subjects with Various Degrees of Renal Function

**DOI:** 10.1093/ofid/ofaf695.1361

**Published:** 2026-01-11

**Authors:** Jianguo Li, Qifeng Shi, Jing Feng, Meng Zhao, Hui Zhao, Anastasia McRoberts, Mitesh Sanghvi, Aramayis Kocharyan, Grigor Mamikonyan, Thomas C Marbury, Dyal Garg, Richard Preston, Meijie Le

**Affiliations:** EVOPOINT BIOSCIENCES USA, INC, Concord, Massachusetts; EVOPOINT BIOSCIENCES USA, INC, Concord, Massachusetts; EVOPOINT BIOSCIENCES USA, INC, Concord, Massachusetts; EVOPOINT BIOSCIENCES USA, INC, Concord, Massachusetts; EVOPOINT BIOSCIENCES USA, INC, Concord, Massachusetts; Pharmaron (Germantown) Lab Services Inc, Germantown, Maryland; Pharmaron (Germantown) Lab Services Inc, Germantown, Maryland; Clinartis, LLC, Hollywood, Florida; Clinartis, LLC, Hollywood, Florida; Orlando Clinical Research Center, Orlando, FL; University of Miami, Miami, Florida; University of Miami, Miami, Florida; EVOPOINT BIOSCIENCES USA, INC, Concord, Massachusetts

## Abstract

**Background:**

Funobactam (formerly XNW4107) is a novel non-β-lactam diazabicyclooctane β-lactamase inhibitor with potent and selective direct activity against Ambler classes of A, C, D β–lactamases. This study evaluated pharmacokinetics (PK), safety, and tolerability of a single intravenous (IV) dose of imipenem 500 mg/ cilastatin 500 mg in combination with funobactam 250 mg or imipenem 200 mg/ cilastatin 200 mg in combination with funobactam 100 mg in subjects with various degrees of renal function.

**Methods:**

Thirty-nine subjects were enrolled into 5 study cohorts with normal renal function with an estimated glomerular filtration rate (eGFR) ≥ 90 mL/min/1.73m^2^ (Cohort 1), mild renal impairment with eGFR ≥ 60 to < 90 mL/min/1.73m^2^ (Cohort 2), moderate renal impairment with eGFR ≥ 30 to < 60 mL/min/1.73m^2^ (Cohort 3), severe renal impairment with eGFR ≥ 15 to < 30 mL/min/1.73m^2^ (Cohort 4) and subjects receiving hemodialysis (Cohort 5). Blood and urine samples were collected to determine funobactam, imipenem and cilastatin concentrations by LC-MS/MS method. The safety and tolerability were assessed by monitoring adverse events, ECGs, vital sign measurements, physical examinations, and clinical laboratory data.

**Results:**

The mean (SD) of PK parameters for funobactam are shown in the table below:

**Table:** 

PK Parameter (units)	Cohort 1 (N=8)	Cohort 2 (N=8)	Cohort 3 (N=8)	Cohort 4 (N=7)	Cohort 5 (N=8)
C_max_ (µg/mL)	15.36 (2.72)	16.7 (2.39)	15.8 (2.45)	6.89 (1.29)	5.18 (0.97)
AUC_0-inf_ (µg*h/mL)	46.7 (7.73)	61.6 (10.8)	106 (26.1)	67.6 (14.9)	180 (34.9)
CL (L/h)	5.52 (1.20)	4.18 (0.75)	2.47 (0.57)	1.55 (0.39)	0.58 (0.11)
V_Z_ (L)	22.9 (3.98)	21.8 (4.54)	20.5 (3.18)	22.2 (3.44)	21.1 (4.01)
t_1/2_ (h)	2.89 (0.11)	3.62 (0.46)	6.05 (1.77)	10.2 (1.95)	25.7 (4.15)
CLr	3.93 (1.12)	3.34 (0.90)	1.82 (0.68)	0.94 (0.37)	NA

**Conclusion:**

Overall, the clearances of funobactam were progressively decreased with the increasing renal insufficiency for subjects with mild, moderate, severe renal impairment, and subjects receiving hemodialysis, and resulted in correspondingly progressive increase in AUC_0-∞_. Dose adjustment could likely be needed in subjects with renal impairment. Administration of funobactam in combination with imipenem and cilastatin was safe and well tolerated in subjects with normal and impaired renal function.

**Disclosures:**

All Authors: No reported disclosures

